# Repetitive Transcranial Magnetic Stimulation in Fibromyalgia: Exploring the Necessity of Neuronavigation for Targeting New Brain Regions

**DOI:** 10.3390/jpm14060662

**Published:** 2024-06-20

**Authors:** Zoran Velickovic, Goran Radunovic

**Affiliations:** 1 Institute of Rheumatology, Resavska 69, 11000 Belgrade, Serbia; velickovic.z@yahoo.com; 2 School of Medicine, University of Belgrade, Dr Subotića 1, 11000 Belgrade, Serbia

**Keywords:** repetitive transcranial magnetic stimulation (rTMS), fibromyalgia, primary motor area (M1), dorsolateral prefrontal cortex (DLPFC), anterior cingulate cortex (ACC), medial prefrontal cortex (mPFC), inferior parietal lobule (IPL), neuronavigation

## Abstract

Fibromyalgia and osteoarthritis are among the most prevalent rheumatic conditions worldwide. Nonpharmacological interventions have gained scientific endorsements as the preferred initial treatments before resorting to pharmacological modalities. Repetitive transcranial magnetic stimulation (rTMS) is among the most widely researched neuromodulation techniques, though it has not yet been officially recommended for fibromyalgia. This review aims to summarize the current evidence supporting rTMS for treating various fibromyalgia symptoms. Recent findings: High-frequency rTMS directed at the primary motor cortex (M1) has the strongest support in the literature for reducing pain intensity, with new research examining its long-term effectiveness. Nonetheless, some individuals may not respond to M1-targeted rTMS, and symptoms beyond pain can be prominent. Ongoing research aims to improve the efficacy of rTMS by exploring new brain targets, using innovative stimulation parameters, incorporating neuronavigation, and better identifying patients likely to benefit from this treatment. Summary: Noninvasive brain stimulation with rTMS over M1 is a well-tolerated treatment that can improve chronic pain and overall quality of life in fibromyalgia patients. However, the data are highly heterogeneous, with a limited level of evidence, posing a significant challenge to the inclusion of rTMS in official treatment guidelines. Research is ongoing to enhance its effectiveness, with future perspectives exploring its impact by targeting additional areas of the brain such as the medial prefrontal cortex, anterior cingulate cortex, and inferior parietal lobe, as well as selecting the right patients who could benefit from this treatment.

## 1. Introduction

**Figure 1:**
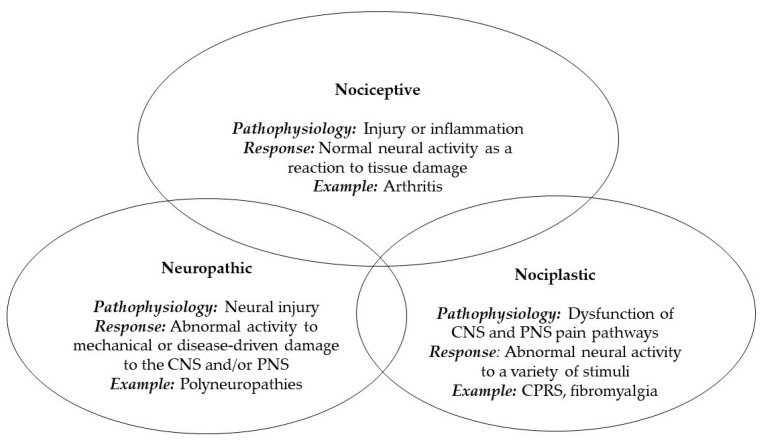
Every patient in rheumatology may experience pain stemming from one or multiple underlying mechanisms. Therefore, it is crucial to view this classification as a spectrum of pain rather than rigidly distinct categories.

Fibromyalgia syndrome (FMS) is a medical condition that predominantly affects young to middle-aged females worldwide. It is characterized by chronic, widespread pain across multiple areas of the body, without discernible evidence of tissue or nerve damage [1]. In some cases, patients with FMS experience pain as their sole symptom, leading to a diminished quality of life and hindered functionality in routine tasks both at home and in the workplace [2,3]. Historically, chronic pain has been categorized into the following two distinct types: nociceptive pain, which stems from prolonged input signaling tissue damage and is typically well-defined and localized by patients (such as in cases of rheumatoid arthritis), and neuropathic pain, resulting from injury or disease affecting the peripheral or central nervous system, frequently observed in conditions like diabetic neuropathy or carpal tunnel syndrome [4]. Nociplastic pain has emerged as a third primary clinical descriptor in the medical lexicon, distinguishing pain disorders that are mechanistically and clinically separate from the aforementioned categories [5]. FMS serves as a prototypical example of nociplastic pain disease, considered within the spectrum that encompasses primary chronic pain syndromes like chronic widespread pain (CWP), nonspecific lower back pain, complex regional pain syndrome (CRPS), chronic primary headaches, and orofacial pain. These conditions typically involve alterations in nociceptive processing mechanisms [6]. However, it is important to note that individuals with rheumatic disorders frequently experience all three forms of pain [7]; therefore, it should not be viewed as an entirely distinct category (Figure 1). 

In FMS, pain is characterized by heightened sensitivity to both painful and non-painful tactile stimuli, along with increased reactivity to environmental triggers such as auditory, visual, and olfactory stimuli. Other distressing symptoms typical of nociplastic pain include stiffness, fatigue, disrupted sleep patterns with non-restorative sleep, cognitive difficulties (referred to as “fibrofog”), and mood alterations like anxiety and/or depression [8]. It is essential for any patient presenting with chronic pain to undergo evaluation for chronic widespread pain (CWP) [9] and the other key symptoms of FMS [10,11], as it can serve as a valuable prognostic tool, aiding in predicting the likelihood of a negative outcome for the patient. This evaluation should include a comprehensive medical history, physical examination, and basic laboratory tests to rule out potential underlying inflammatory conditions. FMS can be difficult to diagnose since no clinical, imaging, or laboratory biomarker exists to confirm or deny its presence [12]. A diagnosis is made if the following criteria are met [10]:

- Generalized pain is present, defined as pain in at least four of the five regions.
- Widespread pain index (WPI) score of ≥ 7 and symptom severity scale (SSS) score of ≥ 5 or WPI of 4–6 and SSS score of ≥ 9.
- Symptoms have been continuous for at least three months.

Fibromyalgia is an acceptable diagnosis regardless of other conditions. A fibromyalgia diagnosis does not rule out the existence of other clinically significant disorders.

It can be difficult for patients to describe their symptoms, and healthcare professionals, as well as patients, may struggle to comprehend the condition’s complexities. FMS is seldom diagnosed in isolation; rather, patients often meet criteria for other primary chronic pain syndromes or mental health disorders [6,13,14], or their symptoms may be associated with various inflammatory musculoskeletal conditions. Intrusive fatigue, which is more cognitive or emotional than physical; hypersensitivity to sound, light, or temperature; and symptom duration and ineffective therapy, both pharmaceutical and non-pharmacological, are some of the diagnostic indicators [10,12].

## 2. Pathophysiology

**Figure 2:**
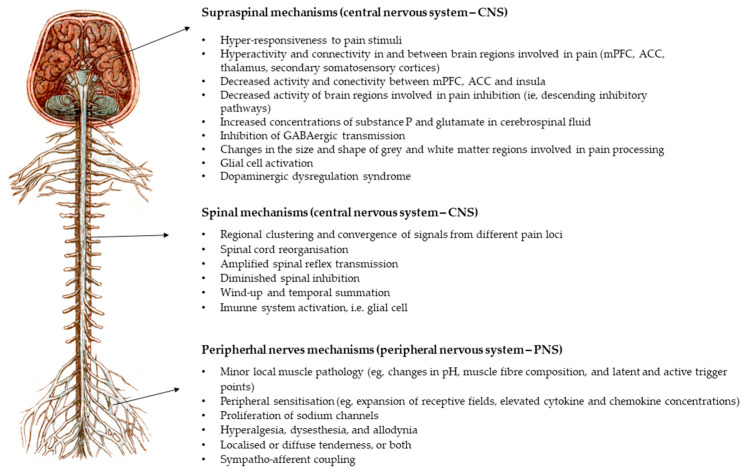
Pathophysiology of fibromyalgia and other nociplastic pain conditions (modified with permission from [4]). Abbreviations: mPFC—medial prefrontal cortex, ACC—anterior cingulate cortex.

Fibromyalgia is characterized by the amplification of nociceptive signaling, formerly referred to as central sensitization. This condition also involves genetic predispositions, alterations in neuroendocrine and autonomic system function, environmental triggers, and cognitive-emotional factors [15]. Contributing factors to fibromyalgia include increased central processing of sensory inputs, now termed central amplification (the bottom-up hypothesis), and/or dysfunctional endogenous pain inhibitory pathways at various levels of the nervous system (the top-down hypothesis) [4]. Persistent nociceptive stimuli are believed to induce functional and morphological changes in the brain and alter control systems, thereby exacerbating the perception of pain. Mechanisms for regulating pain perception exist at supraspinal, spinal, and peripheral levels [16], and the accumulating published data have elucidated changes in each of these levels, either independently or in combination (Figure 2).

Advancements in understanding supraspinal pain processing mechanisms are being made through a range of neuroimaging techniques. These include structural methods like magnetic resonance imaging (MRI) and voxel-based morphometry (VBM) [17], molecular approaches such as magnetic resonance spectroscopy (MRS) and positron emission tomography (PET) [18], and functional imaging modalities such as functional magnetic resonance imaging (fMRI) [19,20]. Through these methods, altered activations in numerous brain regions involved in pain processing have been revealed:

- Prefrontal Cortex (PFC): Involved in executive functions such as decision-making, attention, and emotional regulation. Dysfunction in the PFC may contribute to cognitive symptoms commonly experienced by fibromyalgia patients, such as memory problems, difficulty concentrating, and impaired decision-making in response to pain [21,22].
- Motor Cortex (M1): Research using single- and double-pulsed TMS and fMRI has demonstrated decreased inhibition in the motor cortex, changes in connectivity with other brain regions, and altered motor-evoked potentials (MEPs). These findings are associated with symptoms like pain, stiffness, and impaired motor function. FMS patients exhibit an imbalance in motor cortex regulation, characterized by reduced intracortical inhibition and higher resting motor thresholds (rMTs) compared to healthy controls. This dysfunction can be adjusted through non-invasive brain stimulation, which can enhance intracortical inhibition [23,24].
- Anterior Cingulate Cortex (ACC): The ACC plays a crucial role in pain perception, emotion processing, and the integration of cognitive and affective aspects of pain. Abnormal activation of the ACC is commonly observed in fibromyalgia patients during pain-processing tasks, suggesting alterations in the brain’s response to nociceptive stimuli [22,25].
- Insular Cortex (IC): The IC is involved in processing interoceptive signals, including pain, temperature, and visceral sensations. Dysfunction in the insular cortex may contribute to the hypersensitivity experienced by fibromyalgia patients [26,27].
- Thalamus: The thalamus serves as a relay station for sensory information traveling from the periphery to the cortex. Alterations in thalamic function have been observed in fibromyalgia patients, potentially contributing to abnormal sensory processing and the amplification of pain signals [21,26].
- Amygdala: The amygdala plays a central role in emotion processing, fear conditioning, and the modulation of pain responses. Dysregulation of the amygdala in fibromyalgia patients may contribute to heightened emotional responses to pain stimuli and the development of comorbid mood disorders such as anxiety and depression [22].
- Brainstem: The brainstem contains nuclei involved in pain modulation, including the periaqueductal gray (PAG) and the rostroventromedial medulla (RVM). Dysfunction in brainstem pain-modulatory circuits may contribute to abnormalities in pain inhibition and amplification [28].
- Somatosensory Cortex: The somatosensory cortex is responsible for processing tactile, proprioceptive, and nociceptive information. Altered somatosensory processing in a fibromyalgia patient may result in abnormal pain perception, hypersensitivity to tactile stimuli (allodynia), and exaggerated responses to noxious stimuli (hyperalgesia) [29].

The interconnectedness of these brain regions forms a complex network involved in pain processing, emotion regulation, and cognitive functioning. The default-mode network (DMN) is a network of active brain regions when the mind is at rest and not focused on the outside world or engaged in specific tasks. Dysregulation within this network may contribute to the multifaceted symptomatology observed in fibromyalgia patients [30]. Additionally, PET studies have demonstrated changes in various neurotransmitter systems within the brain, including dopaminergic, serotonergic, GABAergic, and opioid systems [18], each exerting its influence on pain perception [2].

Neuroinflammation, a complex process involving inflammatory responses within the nervous system, has emerged as a potential contributor to the pathophysiology of FMS. While FMS was historically considered a disorder primarily characterized by aberrant pain-processing in the central nervous system, growing evidence suggests that neuroinflammation may play a significant role in the development and perpetuation of FMS symptoms [25,29]. Neuroinflammation encompasses inflammation in both the peripheral nervous system (including nerves and ganglia) and the central nervous system (encompassing the brain and spinal cord) [31], with some researchers even noting chronic systemic inflammation [32]. This inflammation involves changes in blood-vessel function that increase the permeability of the blood–brain barrier, the activation of glial cells (resulting in the release of various mediators), the infiltration and activation of white blood cells, and the heightened production of pro-inflammatory cytokines and chemokines [26]. Bidirectional communication between the nervous and immune systems, known as neuroimmune interactions, plays a crucial role in regulating inflammatory processes within the central nervous system. Dysregulation of these interactions may contribute to the perpetuation of neuroinflammation and the chronicity of FMS symptoms. This evolving understanding of FMS has prompted a shift in perspective, with some researchers suggesting that fibromyalgia could be classified as an autoimmune disease [33]. Despite the growing evidence implicating neuroinflammation in FMS, the precise mechanisms underlying these inflammatory processes and their causal relationship to FMS pathophysiology remain areas of active investigation. Future research efforts aimed at elucidating the role of neuroinflammation in FMS may lead to the development of novel therapeutic approaches targeting inflammatory pathways within the central nervous system, potentially offering new hope for individuals living with this debilitating condition.

## 3. Non-Invasive Repetitive Transcranial Magnetic Stimulation of the Brain

### 3.1. Method Description and Mechanism of Action

Repetitive transcranial magnetic stimulation (rTMS) is a non-invasive neurostimulation technique used to modulate brain activity [34]. Its mechanism of action involves the application of brief magnetic pulses to specific regions of the brain. These pulses can either enhance or inhibit neural activity, depending on various parameters such as frequency, intensity, and duration of stimulation. For instance, a low-frequency rTMS protocol appears to reduce cortical excitability, while a high-frequency protocol tends to increase it [35]. Studies have shown that motor cortex inhibition is a potential biomarker for fibromyalgia [23,24]. rTMS utilizes a coil placed on the scalp, which generates rapidly changing magnetic fields that can penetrate the skull to reach the underlying brain tissue. According to Faraday’s law of electromagnetic induction, these rapidly changing magnetic fields induce electrical currents in the neurons of the targeted brain region that are strong enough to depolarize neurons and trigger action potentials [36].

One key mechanism underlying the therapeutic effects of rTMS is neuroplasticity. Repetitive stimulation induces long-lasting changes in synaptic strength and neuronal connectivity within the stimulated brain regions and their interconnected networks [37]. This neuroplasticity can lead to alterations in cortical excitability, neurotransmitter release, and functional reorganization, potentially underlying the therapeutic benefits observed in various neuropsychiatric disorders [38]. rTMS has been shown to modulate the release and activity of various neurotransmitters, including dopamine, serotonin, gamma-aminobutyric acid (GABA), and glutamate, which play crucial roles in regulating mood, cognition, and behavior. Their dysregulation is implicated in several neuropsychiatric disorders. By modulating neurotransmitter levels and activities, rTMS may restore balance within neural circuits and alleviate the symptoms associated with these disorders. The effects of rTMS are not limited to the directly stimulated brain region but can also influence interconnected brain networks [39], propagating neural activity across these networks to exert widespread effects on cognitive and emotional processes.

### 3.2. Stimulation Protocols and Investigated Outcomes

Different types of rTMS have been studied, each with its own set of stimulation parameters including intensity, frequency, and duration. Furthermore, past research has examined various protocols, taking into consideration factors such as the number of sessions, target areas, and the integration of other interventions (Supplementary Materials).

### 3.3. Frequency

In many studies and clinical applications, rTMS for fibromyalgia typically utilizes frequencies ranging from 1 Hz (low frequency) to 20 Hz (high frequency), with 10 Hz being the most common [40]. 

### 3.4. Number of Sessions

The number of rTMS sessions for fibromyalgia varies depending on several factors including the severity of symptoms, individual response to treatment, and the specific protocol used by the healthcare provider. Typically, a course of rTMS treatment for fibromyalgia consists of 10–15 daily sessions over several weeks, ranging from 2 to 6 weeks, with each session lasting approximately 10 to 30 min [41].

### 3.5. Brain Target Areas

rTMS for fibromyalgia typically targets specific brain regions believed to be involved in pain processing and modulation. While different studies have targeted various brain areas, the most common target regions for rTMS in fibromyalgia include the dorsolateral prefrontal cortex (DLPFC) (left or right) [41] and primary motor cortex (M1) (left or right) [42,43], and two studies have targeted the anterior cingulate cortex (ACC)/medial prefrontal cortex (mPFC) [44,45], with one focusing on a diffuse application of rTMS [46].

### 3.6. Outcomes

When assessing the effectiveness of repetitive transcranial magnetic stimulation (rTMS) in FMS, various clinical outcomes have been utilized [41] (Supplementary Materials).

### 3.7. Evidences for the Therapeutic Use of rTMS in FMS Patients

Following initial systematic review on the efficacy of rTMS in FMS patients [47], we found that there are varied data on this non-pharmacological intervention, especially regarding randomized controlled trials (RCTs) (Supplementary Materials), included in consecutive systematic reviews and meta-analyses. Marlow et al. examined four RCTs, targeting different brain regions such as the left DLPFC [48], right DLPFC [49], and left M1 [50,51]. Their findings suggested that high-frequency rTMS (HFrTMS) on the M1 region could potentially alleviate pain but did not demonstrate significant effects on depression or overall quality of life. Four years later, Knijnik et al. [52] presented another meta-analysis and systematic review by adding two additional RCTs, targeting the left M1 [53], right DLPFC (low-frequency rTMS), and left M1 (HFrTMS) regions [54]. Their analysis revealed a statistically significant enhancement in quality of life measured through the fibromyalgia impact questionnaire (FIQ), with no effect on pain and depression. 

During this period, three more studies were conducted utilizing HFrTMS over the left M1 region [55] and left DLPFC [56], with the diffuse application of low-intensity HFrTMS [46]. Saltychev and Laimi summarized these new studies along with older ones, focusing solely on pain reduction as the outcome [57]. The findings suggested that rTMS demonstrated similar effectiveness to sham treatments in reducing pain severity among fibromyalgia patients, raising questions about conventional supports for this method in fibromyalgia management. 

Simultaneously, Hou et al. [58] conducted a meta-analysis examining the impacts of non-invasive brain stimulation (NBS), encompassing rTMS and transcranial direct current stimulation (TDCS). Their analysis incorporated RCTs involving LFrTMS [59] and HFrTMS [60] over the left M1. Nevertheless, they concluded that NBS demonstrated efficacy across various domains in FMS patients, encompassing pain, depression, fatigue, sleep, tender points, and overall health/function, and that the favorable effects between TDCS and rTMS generally align. 

**Figure 3:**
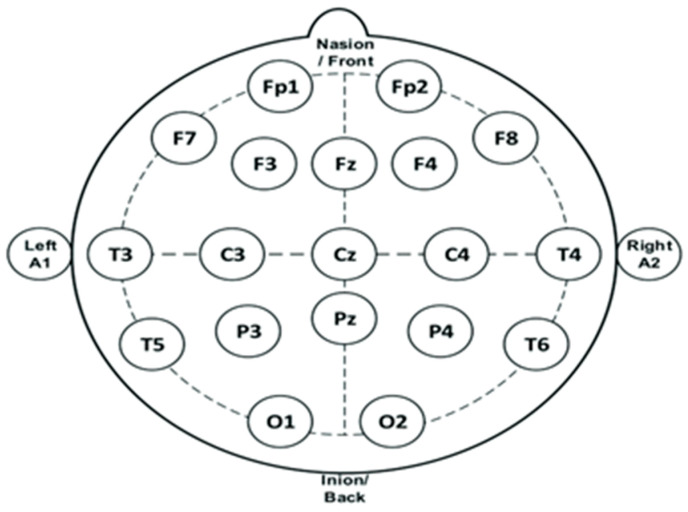
An illustration of the 10–20 system initially used for the neuronavigation of targeted brain regions. F3—left DLPFC, F4—right DLPFC.

The next systematic review about NBS in FMS patients was conducted by Conde-Antón and colleagues in 2020 [61], with two new RCTs on HFrTMS targeting brain regions of the left DLPFC [62], left M1, and left DLPFC [63] using an EEG 10–20 system for neuronavigation (Figure 3).

**Figure 4:**
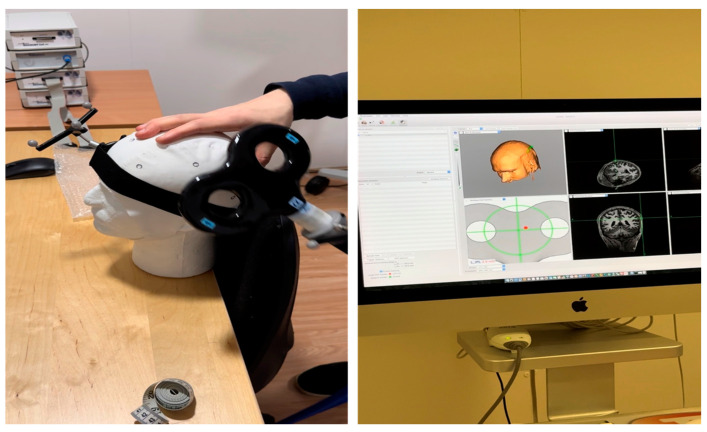
Example of the MRI-guided placement of a magnetic coil over the left IPL (with the kindness of Prof. Dr. Neil Basu from the University of Glasgow).

Their findings indicated that applying rTMS to the M1 region can alleviate short- and medium-term pain for individuals with FMS. However, the results regarding mood changes, quality of life, and fatigue were inconsistent. New randomized controlled trials (RCTs) emerged in 2019, 2020, and 2021. Cheng and colleagues [64] utilized MRI scans and brain-navigation computer software to precisely and consistently direct a coil to the region of interest. This was followed by studies by Bilir et al. [65] and Guinot et al. [66] (Figure 4).

In contrast, two other RCTs by Tanwar et al. [67] and Izquierdo-Alventosa et al. [68] did not employ such neuronavigation techniques. All of these studies have contributed to a systematic review and meta-analysis conducted by Su et al. of 18 RCTs [69]. This meta-analysis demonstrated the safety and efficacy of rTMS in treating various aspects of fibromyalgia symptoms, including quality of life, pain, and, notably, for the first time, depression and anxiety. Systematic reviews and meta-analyses released in 2022 examined similar sets of RCTs as previous studies. The overall consensus is that HFrTMS targeting the left M1 has an analgesic effect and positively impacts quality of life [42,70,71], while Sun et al. [72] argued that LFrTMS over the DLPFC might offer a superior solution with the same outcomes. Zhu et al. [40] evaluated the effectiveness of HFrTMS (10 Hz) and demonstrated that stimulation of either brain region—the DLPFC or M1—could alleviate pain levels. 

A recently published paper by Martinez et al. [41] summarized 11 systematic reviews and meta-analyses previously described. The primary objective of this umbrella and mapping review was to consolidate evidence regarding the impact of rTMS on alleviating pain intensity, depressive symptoms, anxiety, and overall health in comparison to sham rTMS interventions among FMS patients. The findings indicated that high-frequency rTMS protocols, when applied over the M1, demonstrated a noteworthy reduction in pain intensity lasting for at least one month of follow-up, whereas such effects were not observed when rTMS was applied over the DLPFC. Moreover, irrespective of the protocol and area of application, rTMS did not exhibit significant efficacy in alleviating depressive and anxiety symptoms. However, concerning overall health, both high- and low-frequency rTMS protocols led to notable improvements post-intervention. This latter finding was the most consistent among the reviewed systematic reviews. Chamizo et al. [73] and Cheng et al. [74] conducted systematic reviews on rTMS for FMS, arriving at conclusions akin to previous research. They also incorporated a recent RCT comparing rTMS and transcranial direct current stimulation (TDCS) but noted that this trial lacked a sham control [75]. All of the aforementioned systematic reviews and meta-analyses are summarized in Table 1.

One study examined the use of rTMS in patients with fibromyalgia but lacked a sham control group, instead comparing it to prolotherapy [76]. Although recent meta-analyses have included numerous randomized controlled trials (RCTs), they have not incorporated the high-quality RCT conducted by Argaman in 2021 [43], which specifically targeted the right M1 brain region for the first time, and as described in the literature, Argaman’s study revealed significant outcomes, showing marked reductions in MPQ-VAS and BPI-severity scores, as well as decreases in FIQ and MPQ-affective scores following 10 Hz rTMS. Unfortunately, we were not able to obtain the paper by Lacroix et al. [77], who noted a significant clinical improvement with rTMS treatment after the induction phase, maintained for six months, particularly with regard to the PGIC measure of pain, as well as reductions in fatigue and depression intensity. A recent RCT by Badr [78] demonstrated the potential efficacy of LFrTMS over the right DLPFC in significantly impacting depression, anxiety, functionality, and cognition over both short- and long-term periods. A novel study by Tiwari et al. [79] demonstrated that neuro-navigated LFrTMS over the DLPFC was effective in managing pain, as well as cognitive and sleep disturbances, in patients with fibromyalgia. A recently published study by Kankane et al. [80] demonstrated that both LFrTMS over the right DLPFC and HFrTMS over the left DLPFC were effective and safe for the management of pain, depression, and anxiety, thereby improving the quality of life in patients with FMS compared to sham treatments. In the study by Tilbor et al. [45], a new target brain region, the ACC/mPFC, was stimulated with a frequency of 20 Hz, proposing a new stimulation protocol for deep TMS (dTMS). They proposed an innovative hypothesis that the effectiveness of pain-focused psychotherapeutic interventions is not solely attributed to dTMS but rather an effect of dTMS-induced plasticity in pain-related networks.

It is noteworthy that all of these studies used numerical clinical scales. However, two studies by Argaman et al. [24,43] took a more personalized approach by searching for potential biomarkers. They utilized fMRI to investigate whether MRI-based resting-state functional connectivity (rsFC) could predict the response to M1-rTMS. They found that with FMS, a weaker initial connection between pain-related brain regions and networks (such as the default-mode network, middle frontal gyrus, rostro-medial prefrontal cortex, thalamus, pregenual anterior cingulate cortex, inferior parietal lobule, anterior insula, and angular gyrus) is associated with greater pain relief from M1-rTMS. Another study by Jung et al. [39] also sought to explain the mechanism of action of M1-rTMS. Therefore, while this stimulation protocol is effective, selecting the right patients who could benefit from this treatment remains a challenging issue that needs to be addressed.

### 3.8. Limitations of rTMS

While rTMS is promising for FMS patients, it is not without limitations. In clinical trials, accurately assessing the effectiveness of rTMS can be complicated by the placebo effect, which is particularly relevant for FMS patients who are prone to it. Although sham stimulation is commonly used for a control, its ability to effectively blind participants is not always guaranteed. Additionally, rTMS primarily influences surface-level cortical regions, potentially limiting its reach to the deeper brain structures involved in pain networks. Moreover, individual responses to rTMS can vary significantly, making it difficult to predict its efficacy for specific patients. Furthermore, the effects of rTMS tend to be temporary, necessitating repeated sessions to sustain therapeutic benefits, which can inconvenience patients. While generally safe, rTMS can induce mild side effects like headaches, scalp discomfort, and muscle twitching. In rare instances, it may trigger seizures, especially in individuals with a history of epilepsy. Access to rTMS therapy may be constrained by its cost and the need for specialized equipment and trained personnel, posing challenges to widespread adoption, particularly in resource-limited areas. Long-term data on the efficacy and safety of rTMS for FMS remain limited, necessitating further research to comprehensively understand its potential advantages and risks over extended periods. Lastly, certain medical conditions, such as implanted metallic devices in the head, a history of seizures, or specific psychiatric disorders, may contraindicate rTMS usage. Proper screening for these contraindications is crucial to ensure patient safety.

### 3.9. FMS Treatment Recommendations

The principles of general management and initial treatment guidelines for FMS from Israel (2012) [81], Canada (2012) [82], Germany (2012) [83], and the European Alliance of Associations for Rheumatology (EULAR) (2016) [84] advocate for activities like aerobic and strengthening exercises. While techniques such as meditative movement therapies, mindfulness-based stress reduction, acupuncture, and hydrotherapy receive less robust recommendations, treatments like hypnosis, massage, and complementary and alternative therapies have not been endorsed [85]. Psychological interventions, particularly cognitive behavioral therapy (CBT), are recommended, especially for individuals with mood disorders or coping challenges, with strong endorsement in the German 2012 guidelines [83]. Despite advancements in understanding FMS pathogenesis, pharmaceutical treatments lack strong support in these guidelines. If used, they are suggested as supplements to non-pharmacological interventions. Medications such as duloxetine, tramadol, amitriptyline, cyclobenzaprine, and pregabalin are mentioned for severe pain and sleep disturbances, with advice to discontinue if no improvement is observed within a reasonable timeframe. It is emphasized that treatment plans should be individualized, taking into consideration medication risks and adverse effects that could complicate the clinical picture [83]. Multimodal rehabilitation programs are recommended for individuals with severe impairments, although current data supporting their effectiveness are limited. Overall, a graded approach to improving quality of life is advocated. 

Recent recommendations, such as those from Italy in 2021 [86] and international guidelines [87], continue to emphasize aerobic exercise, education, sleep hygiene, and CBT as core treatments for all FMS symptoms. Additional interventions have gained consensus as adjunctive treatments for specific symptoms. However, it is noteworthy that despite evolving evidence regarding their efficacy, non-invasive brain stimulation therapies are not mentioned in any form in these guidelines. This absence suggests an area warranting further exploration and consideration in future updates to FMS management guidelines, particularly as research progresses in this domain.

### 3.10. Future Perspectives

Recently published studies on the pathophysiology of FMS have suggested a potential new target region for rTMS. Specifically, targeting the left inferior parietal lobe, a surface region of the brain, with rTMS has emerged as a promising approach for modulating cognitive, sensory, and motor functions in FMS patients [88,89]. However, it is worth noting that this area is considerably smaller than the more commonly targeted regions like the M1 and DLPFC, necessitating precise targeting methods. The use of neuronavigation technology, which integrates neuroimaging data such as MRI scans with the rTMS procedure, is essential. This allows for the accurate placement of the stimulation coil over the target region, enhancing the precision and reliability of the stimulation. By employing neuronavigation, clinicians can optimize treatment outcomes for FMS patients while minimizing the risk of adverse effects associated with off-target stimulation. This approach further emphasizes the importance of personalized, neuroanatomically guided rTMS interventions in select FMS patients. 

## 4. Conclusions

An extensive review of RCTs, systematic reviews, and meta-analyses, along with one mapping review, has offered compelling evidence endorsing the effectiveness of HFrTMS directed at the left M1 for alleviating pain and improving quality of life in FMS patients. However, it is important to note that none of the studies outlined a standardized stimulation protocol, mainly due to the significant heterogeneity observed across the RCTs. The inconsistency in stimulation protocols could hinder the integration of this modality into new treatment guidelines. A more personalized approach is necessary to identify the right patients who can benefit from this treatment. Addressing the method’s limitations through ongoing research with more objective outcomes, technological advancements, and refining treatment protocols and target regions can improve the clinical utility of rTMS for selected FMS patients.

## Figures and Tables

**Table 1:** Summarized conclusions from systematic reviews (SR) and meta-analyses (MA).

Authors (by Year)	No. of RCTs	Conclusion
Marlow et al. 2012 [47]	4	Pain reduction and improved quality of life/general health (measured by FIQ) were observed with HFrTMS over the left M1, but there were no changes in depressive symptoms. The effects of stimulating the right or left DLPFC on these outcomes remain inconclusive.
Knijnik et al. 2015 [52]	5	In comparison with sham stimulation, rTMS demonstrated a superior effect on the quality of life in patients with FMS 1 month after starting therapy, but the brain region was not specified. These statistically significant changes were not found in depression or pain intensity, irrespective of the target area (left M1 or left or right DLPFC).
Hou et al. 2016 [58]	11	The general conclusion was that rTMS could have an influence on multiple domains in FMS patients, irrespective of the target area. rTMS over the left M1 may better reduce pain, and the stimulation of the DLPFC may improve depression. No clear conclusion can be obtained from this paper since both rTMS and transcranial direct current stimulation (tDCS) were evaluated.
Saltychev and Laimi 2016 [57]	8	The meta-analysis focused solely on the following measure: is there a change in pain severity? It provides moderate evidence suggesting that rTMS is not superior to sham treatment in alleviating pain severity in fibromyalgia patients, regardless of the targeted brain area. However, its impact on other outcomes was not assessed.
Conde-Antón et al. 2020 [61]	8	The findings indicated that HFrTMS over the left M1 significantly impacted pain intensity and overall health in FMS patients, though it did not affect depressive and anxiety symptoms. Additionally, stimulation of the DLPFC showed no significant effects on any of the measured outcomes.
Su et al. 2021 [69]	18	Subgroup analysis (by stimulation site, M1 or DLPFC) showed that rTMS over the M1 area was effective in improving quality of life (FIQ/FIQR score), reducing pain intensity (BPI interference subscale score and MPQ score), and improving depression (BDI score), and rTMS over the DLPFC reduced the FIQ/FIQR score, pain intensity (MPQ score, number of tender points), and depressive symptomatology (HDRS score). Anxiety was improved (evidenced by the HADS scores), and there was no influence on fatigue (FSS score). However, no significant differences were detected between subgroups for any outcomes as the researchers did not specify the target area for each outcome.
Kim et al. 2021 [42]	5	The results showed statistically significant results regarding general health but not pain intensity and depressive symptoms in patients with FMS. Only RCTs with M1 as the target area were evaluated.
Choo et al. 2022 [70]	10	The following outcomes were evaluated: pain, depression, anxiety, and general health. HFrTMS over the M1 had a positive pain-reducing effect immediately, and the patient’s general health improved after 5–12 weeks. However, DLPFC stimulation was not effective in controlling any of the fibromyalgia symptoms.
Sun et al. 2022 [72]	14	The results showed that rTMS relieved pain and enhanced the general health of patients with FMS; however, on the basis of current reports, it did not improve anxiety and depression. Subgroup analysis (HFrTMS over the M1, LFrTMS over the M1, HFrTMS over the DLPFC, and LFrTMS over the DLPFC) showed that LFrTMS in the DLPFC region is the optimal protocol for relieving pain.
Toh et al. 2022 [71]	11	rTMS is more effective than sham in improving pain and quality of life, but it did not demonstrate reductions in depression or anxiety in patients with FMS. Subgroup analysis of the stimulation site showed that M1 stimulation was more effective than sham stimulation in improving quality of life and pain reduction compared to DLPFC stimulation.
Chamizo et al. 2023 [73]	7	The pain-reducing capacity of rTMS when applied over the left M1 was accompanied by an improvement in quality of life. Targeting the left DLPFC yielded moderate impacts on pain intensity, fatigue, and depression. Stimulating the dACC led to decreased pain intensity.
Zhu et al. 2023 [40]	7	HFrTMS (10 Hz) had significant effects on analgesia and improved general health in patients with FMS but did not improve depression. A subgroup analysis of pain reduction based on stimulation at the M1 and DLPFC showed no significant difference.
Cheng et al. 2024 [74]	13	HFrTMS over the M1 led to significant pain reduction and improvement in QoL.
Martinez et al. 2023 [41]	11 MA and SR	The results showed that HFrTMS applied on the M1 showed some effect on pain intensity, with a limited quality of evidence. rTMS was shown to be effective in improving general health, with a moderate quality of evidence (irrespective of target area). Finally, rTMS was not shown to be effective in managing depressive symptoms and anxiety, with a limited to moderate quality of evidence.

## Data Availability

All data are available in the manuscript and in Supplementary Materials.

## Supplementary Materials

The following supporting information can be downloaded at https://www.mdpi.com/article/10.3390/jpm14060662/s1: Table S1, List of clinical trials regarding repetitive trancranial magnetic stimulation in fibromyalgia patients. References [44,45,48,49,50,51,53,54,55,56,59,60,62,63,64,65,66,67,68,78,80,90,91] have been cited in the Supplementary Materials.
