# Supplementary material for: Repetitive Transcranial Magnetic Stimulation in Fibromyalgia: Exploring the Necessity of Neuronavigation for Targeting New Brain Regions
Source: J Pers Med. 2024 Jun 20;14(6):662. doi: 10.3390/jpm14060662 (PMC11204413; doi:10.3390/jpm14060662)
Supplement: Supplementary file 1 [file jpm-14-00662-s001.zip › jpm-3038081.pdf]

Table S1. List of clinical trials regarding repetitive trancranial magnetic stimulation in fibromyalgia patients.

Study	Authors	Active/Sham	Frequency	Target region	Navigation	Pain	QoL	Depression	Anxiety	Fatigue	Sleep	Catastrophizing	Cognition
**RCT**	Passard 2007 [51]	13/13	10 Hz	L M1	TMS/MT/ MEP	BPI, MGPQ, PPT	FIQ	HDRS, BDI-II, HADS	HADS				
**RCT**	Carretero 2009 [49]	14/12	1 Hz	R DLPFC	TMS/MT/ MEP	LPS	CGI	HDRS, BDI-II		ZFFC			
**RCT**	Mhalla 2011 [50]	16/14	10 Hz	L M1	TMS/MT/ MEP	BPI, MGPQ	FIQ	HADS, BDI-II				PCS	
**RCT**	Short 2011 [48]	10/10	10 Hz	L DLPFC	TMS/MT/ MEP	BPI	FIQ	HDRS					
**RCT**	Lee 2012 [54]	5/5/5	1 Hz / 10 Hz	R DLPFC/ L M1	TMS/MT/ MEP	VAS, TP	FIQ	BDI-II					
**RCT**	Baudic 2013 [60]	20/18	10 Hz	L M1	TMS/MT/ MEP	BPI		HADS	HADS		MOS-SF-12		Cognition tests
**RCT**	Tekin 2013 [55]	27/24	10 Hz	L M1	TMS/MT/ MEP	VAS	WHOQOL-BREF	MADRS					
**RCT**	Tzabazis 2013 [44]	NA	1 Hz / 10 Hz	dACC	TMS/MT/ MEP	BPI, NRS	FIQ	BDI-II					
**RCT**	Maestu 2014 [46]	28/26	8 Hz	Diffuse	EEG 10-20	PPT, NRS	FIQ	NRS	NRS	NRS	NRS		
**RCT**	Boyer 2014 [53]	16/13	10 Hz	L M1	TMS/MT/ MEP	VAS, TP, PPT	FIQ, SF-36	BDI-II, HADS	HADS				Cognition tests
**RCT**	Yagci 2014 [59]	12/13	1 Hz	L M1	TMS/MT/ MEP	VAS	FIQ	BDI-II					
**RCT**	Avery 2015 [56]	7/11	10 Hz	L DLPFC	TMS/MT/ MEP	NRS, BIRS, BURS, MGPQ, BPI, PPT, TP, VAS-Pain	SF-36, VAS,	HDRS, BDI-II		MFI, VAS-Fatigue	VAS-Sleep		
**RCT**	Fitzgibbon 2018 [62]	11/11	10 Hz	L DLPFC	EEG 10-20	BPI, MGPQ, NRS	SF-36, FIQ	BDI-II	BAI	MFI		PCS	
**RCT**	Altas 2019 [63]	10/10/10	10 Hz	R DLPFC/ L M1	EEG 10-20	VAS	FIQ, SF-36			FSS			
**RCT**	Cheng 2019 [64]	9/10	10 Hz	L DLPFC	MRI	VAS		HDRS					
**RCT**	Tanwar 2020 [67]	45/41	1 Hz	R DLPFC	TMS/MT/ MEP	NRS, MGPQ		HDRS	HARS				
**RCT**	Bilir. 2021 [65]	10/10	10 Hz	L DLPFC	MRI	VAS	FIQ	HADS	HADS	FSS			ACE-R
**RCT**	Guinot 2021	17/19	10 Hz	L M1	MRI	VAS	FIQ	BDI-II			PSQI	PCS	

**RCT**	Izquierdo-Alventosa 2021 [68]	17/16	10 Hz	L M1	TMS/MT/ MEP	VAS, PPT,	FIQ	BDI-II	HAD S	CR-10 Borg scale		
**RCT**	Argaman 2022 [43]	16/19	10 Hz	R M1	TMS/MT/ MEP	BPI, MGPQ	FIQ, SF36	BDI-II	STAI			
**Non-RCT**	Gomez-Arguelles 2022 [90]	N.A	8 Hz	Diffuse	N.A.		CGI				PSQI	
**Non-RCT**	Pareja 2022 [91]	N.A	8 Hz	Diffuse	N.A.	WPI	FIQ					
**RCT**	Badr 2024 [78]	21/21	1 Hz	R DLPFC	TMS/MT/ MEP		FIQ	HDRS	HAR S			MoCa, cogni-tion tests
**RCT**	Tilbor 2024 [45]	8/11	20 Hz	dACC -mPFC	TMS/MT/ MEP	MGPQ, BPI	FIQ	HDRS				
**RCT**	Kankane 2024 [80]	30/30/30	10 Hz/1 Hz	L DLPFC / R DLPFC	TMS/MT/ MEP	NRS	FIQ	HDRS	HAR S			

Abb. L – Left, **R** – Right, **RCT** – randomized clinical trial, **M1** – primary motor cortex, **DLPFC** - dorsolateral prefrontal cortex, **dACC** - dorsal anterior cingulate cortex, **mPFC** – medial prefrontal cortex, **TMS/MT/MEP** – trancranial magnetic stimulation/motor threshold/motor evoked potential, **BPI** - Brief Pain Inventory, **MGPQ** - McGill Pain Questionnaire, **PPT** - Pressure Pain Thresholds, **LPS** - Likert Pain Scale, **TP** - Tender Points, **NRS** - Numeric Rating Scale, **BIRS** - Gracely Box Intensity Scale, **BURS** - Gracely Box Unpleasantness Rating Scales, **WPI** - Widespread Pain Index, **FIQ** - Fibromyalgia Impact Questionnaire, **CGI** - Clinical Global Impression, **SF-36** - 36-Item Short Form Health Survey, **WHOQOL-BREF** - World Health Quality of Life-BREF, **HDRS** - Hamilton Depression Rating Scale, **BDI** - Beck Depression Inventory, **MADRS** - Montgomery Asberg Rating Scale, **HADS** - Hospital Anxiety and Depression Scale, **BAI** - Beck Anxiety Inventory, **BDI – II -** BDI - Beck Depression Inventory Second Edition, **HARS** - Hamilton Anxiety Rating Scale, **STAI** - State-Trait Anxiety Inventory, **ZFFC** - Zachrisson FibroFatigue Scale, **MFI** - Multidimensional Fatigue Inventory, **FSS** - Fatigue Severity Scale, **PSQI** - Pittsburgh Sleep Quality Inventory, **PCS** - Pain Catastrophizing Scale, **ACE-R** - Addenbrooke's Cognitive Examination Revised, **MoCa** – Montreal Cognitive Assessment.
